# Editorial: New insights into pathophysiology and management of pregnancy in systemic autoimmune diseases: Toward new therapeutic approaches

**DOI:** 10.3389/fphar.2022.1017067

**Published:** 2022-10-06

**Authors:** Alessandra Bettiol, Guilherme Ramires de Jesús, Isabell Haase, Laura Andreoli, Maria Letizia Urban

**Affiliations:** ^1^ Department of Experimental and Clinical Medicine, University of Florence, Florence, Italy; ^2^ Department of Obstetrics, Universidade do Estado do Rio de Janeiro, Rio de Janeiro, Brazil; ^3^ Department of Obstetrics, Instituto Fernandes Figueira - FIOCRUZ, Rio de Janeiro, Brazil; ^4^ Policlinic and Hiller Research Unit for Rheumatology, Heinrich Heine University Duesseldorf, Duesseldorf, Germany; ^5^ Department of Clinical and Experimental Sciences, University of Brescia, Brescia, Italy; ^6^ Unit of Rheumatology and Clinical Immunology, ASST Spedali Civili di Brescia, Brescia, Italy

**Keywords:** pregnancy, obstetric complication, autoimmune disease, DMARD (disease modifying anti-rhuematic drugs), Research Topic

Systemic autoimmune diseases (SAD) commonly affect women during childbearing age, representing a clinical challenge in terms of obstetric counseling and pregnancy management. These diseases can be associated with an increased risk of fetal and maternal complications and mortality. On the other hand, pregnancy can remarkably impact disease activity.

In recent years, a growing number of studies have investigated the implications of different immune and non-immune players (such as hormonal changes and microbiomes) on pregnancy outcomes, although the physio-pathogenic mechanisms are not yet fully understood. Despite several attempts being made to improve prophylactic and therapeutic strategies for obstetric complications in SAD, the clinical management in the periconceptional, gestational, and postnatal periods in these conditions is still a matter of debate.

This Research Topic includes contributions about several autoimmune diseases and different aspects related to reproduction and pregnancy, yielding novel insights and future research perspectives.

Among SAD, rheumatoid arthritis (RA) is a relatively frequent disease whose onset can occur during childbearing age. Similarly to other SAD, RA can be negatively influenced by pregnancy, in terms of disease course and flares. In this Research Topic, Gerardi et al. publish a prospective study on 73 pregnant women affected by RA, reporting an incidence of flares during pregnancy of 37%, mainly during the first and second trimesters. Flares occurred more frequently after the discontinuation of biologic disease-modifying anti-rheumatic drugs (bDMARDs) after a positive pregnancy test and were in turn associated with a higher occurrence of preterm delivery.

These findings suggest that women with aggressive RA receiving bDMARDs should be considered for continuing therapy during pregnancy to reduce the risk of flares and adverse pregnancy outcomes.

As drugs have been discontinued during pregnancy in women with inflammatory rheumatic diseases, little is known about whether the same efficacy can be expected upon postpartum resumption. Tahmasian et al. analyze this question for patients with joint inflammatory diseases in the PreCARA cohort. The survival of bDMARDs and methotrexate was analyzed in 63 patients after restarting their medication. Overall, one year-survival after restarting was high, with 85% for methotrexate, and 89% for biologics. Although slightly lower than in a matched, retrospective non-pregnant control group, the data indicate good effectiveness and reassure patients and physicians.

Antiphospholipid antibody syndrome (APS) is another autoimmune condition characterized by recurrent obstetric complications. Within this Research Topic, Hoxha et al. conduct a systematic literature review of 21 studies, summarizing current evidence on additional therapy protocols for preventing recurrent losses in refractory and/or high-risk APS pregnancies. Overall, hydroxychloroquine 200–400 mg and plasma exchange weekly with intravenous immunoglobulin 2 gr/kg/monthly could be taken into consideration in the management of refractory APS and high-risk/refractory APS, respectively.

In women of childbearing age, APS is sometimes detected only due to pregnancy complications and is then defined as obstetric APS (oAPS). The extent to which the risk of thrombotic events in patients with primary oAPS differs from that in patients with primary thrombotic APS (tAPS) is studied in more detail by Niznik et al. In a retrospective cohort study with a follow-up of >10 years in 219 patients, they find a higher rate of new thrombotic events in the tAPS group (50% vs. 35.8%, *p* = 0.06), whereas these patients developed obstetric complications less frequently (10.5% vs. 31.3%, *p* < 0.001). Heart valve disease and ANA positivity were related to thrombosis following a diagnosis of oAPS. Although these results point to differences in the pathogenesis of tAPS and oAPS that are not yet fully understood, the authors conclude that there is nevertheless a substantial risk for thromboembolic events in patients with oAPS. These results might help to shape future guidelines and optimize the prevention of fatal events.

APS and systemic lupus erythematosus are traditionally associated with complement activation, which can account for an increased risk of obstetric complications. Appropriate function of the complement system is necessary for the development of a healthy pregnancy, while complement activation has been linked to pregnancy loss, preeclampsia, and preterm birth. The review by Cavalli et al. discusses the role of the complement system in pregnancy, the mechanisms by which its activation by both classical and alternative pathways can affect gestational outcomes, and how it should be measured. Moreover, they also evaluate the complement system in other rheumatic diseases, such as RA, Sjögren’s syndrome, systemic sclerosis, and vasculitides. The authors conclude that the complement system is an attractive candidate biomarker to stratify the obstetric risk among women with SAD.

Another condition that has limited information during pregnancy is undifferentiated connective tissue disease (UCTD). Serena et al. review the most recent literature and raise some concerns, such as the possibility of severe disease and progression to definite SAD. A high incidence of obstetric complications was reported in patients with UCTD compared to healthy women and special attention should be given to congenital heart block in anti-Ro/SSA positive patients. Similarly to other SAD, pregnancy planning is important to minimize both fetal and maternal complications.

Beyond systemic diseases, organ-specific autoimmune diseases can also negatively affect pregnancy. The impact of autoimmune thyroiditis on pregnancy outcomes has raised great interest in the past few years. Botta et al. add a contribution to the field by collecting a large cohort of more than one thousand healthy pregnant women and show that the frequency of positive anti-thyroid antibodies (anti-thyroid peroxidase antibodies and/or anti-thyroglobulin antibodies) was nearly 5.5%. This frequency was significantly higher (17.5%) in 268 pregnant women with systemic autoimmune diseases. Among these patients, the presence of positive anti-thyroid antibodies was significantly associated with an earlier gestational week at delivery and lower birth weight. This large prospective study suggests that anti-thyroid antibodies should be implemented into the obstetric risk stratification of women with systemic autoimmune diseases who wish to plan a pregnancy.

Notably, SAD can also be associated with gynecological complications outside the pregnancy period. Orlandi et al., within the frame of a multidisciplinary collaboration between rheumatologists and gynecologists, investigate menstruation-related disorders and their impact on the quality of life of women with rheumatic diseases using a self-administered questionnaire. Several disturbances, including heavy menstrual bleeding and gynecological pain, were significantly more frequent in patients as compared to healthy controls. Both physical and mental domains in the short-form 12-item questionnaire were negatively affected by menstruation-related disorders in women with rheumatic diseases. This study highlights the importance of asking patients about these disturbances and promptly referring them to a gynecologist for multidisciplinary management.

Taken together, the eight manuscripts collected in the present Research Topic provide new insights into the pathophysiology and management of pregnancy in SAD. Data from these studies clearly indicate that the times in which women with SAD were discouraged from considering pregnancy are behind us. Nevertheless, pregnancy planning and close interdisciplinary management appear of fundamental importance to prevent both maternal and fetal complications. Despite the recent advancement in this field, there are still many open issues that should be addressed to optimize periconception, gestational and postnatal management in these conditions.

